# Results of the requirement analysis as part of the Austrian research project linked care

**DOI:** 10.1093/eurpub/ckac131.291

**Published:** 2022-10-25

**Authors:** G Salomon, N Sturm

**Affiliations:** Research, Johanniter Ausbildung und Forschung gem. GmbH, Vienna, Austria

## Abstract

Sufficient information is the foundation for efficient and high-quality health care services. In the field of extramural health care, this challenge is particularly evident: missing or insufficient information can lead to underuse, overuse, or misuse of health care, e.g. due to the necessity of multiple assessments or lack of relevant information. The noticeable shortage of health care professionals further underscores the urgent need for efficient and quality-assured health care services. The aim of the project LICA - linked care - is to provide a platform for better coordination and information exchange between health professionals involved in home care, with a focus on ICT in nursing in Austria. The project is funded by the Austrian Research Promotion agency (FFG) as part of the “benefit - demografischer Wandel als Chance” program (April 2020-March 2025). The requirement analysis was conducted from April to December 2021. In light of the user-centered approach a mix of methods was chosen, consisting of: literature analysis, 5 guideline-based focus group interviews, guideline-based expert interviews n = 44 (people in need of care n = 23, health professionals n = 21), documentation-analysis (4 care documentation systems - from participating project partners) and working diaries: n = 5 on 5 consecutive working days (=25 diaries). Therefore, three main target groups were identified: i) people in need of care, ii) healthcare professionals and iii) healthcare providers. The data were analyzed using a qualitative content analysis based on Kuckartz. The main results regarding the status quo are: i) different documentation systems are utilized, ii) lack of digitized documents, iii) currently no standardized documentation system is in use. Thus, following requirements could be identified: i) interoperability with existing systems, ii) setting comprehensive function, iii) usability, iv) interdisciplinary readability, v) error-management, and vi) proper data protection measures.

**Key messages:**

• Healthcare systems are under great pressure worldwide. Innovative solutions can help maintain and improve the quality and efficacy of healthcare services.

• Gapless and efficient information provision is a key essential to high quality healthcare. The project offers a user-centric approach to develop a platform for connectivity of existing systems.

